# Efficacy and safety of BTK inhibitors in Richter’s transformation: a systematic review of clinical evidence

**DOI:** 10.3389/fonc.2025.1681589

**Published:** 2026-01-20

**Authors:** Canan D. Dirican, Folasade Ajayi, Anas Al Mardini, Bolivia Crocete Aloysia Fernandes, Amara Sofia, Venkatesh Gondhi, Hamid Shaaban, Michael Maroules

**Affiliations:** 1 NYMC at Saint Mary’s and Saint Clare’s Internal Medicine Residency Program, Denville, NJ, United States; 2 Saint Michaels Medical Center Division of Hematology and Medical Oncology, Newark, NJ, United States; 3 Saint Mary’s General Hospital Division of Hematology and Medical Oncology, Passaic, NJ, United States

**Keywords:** acalabrutinib, Bruton’s tyrosine kinase inhibitors, CLL, DLBCL, ibrutinib, pirtobrutinib, Richter’s transformation, systematic review

## Abstract

**Background:**

Richter’s transformation (RT) is an aggressive progression of chronic lymphocytic leukemia (CLL) or small lymphocytic lymphoma (SLL), most commonly to diffuse large B-cell lymphoma (DLBCL). Therapeutic options are limited, and outcomes are poor, particularly in relapsed or refractory cases. Bruton’s tyrosine kinase (BTK) inhibitors have transformed the treatment landscape of CLL, but their role in RT is less well defined.

**Methods:**

We conducted a systematic review in accordance with PRISMA guidelines to evaluate the efficacy and safety of BTK inhibitor–based therapies in patients with RT. PubMed, EMBASE, and ClinicalTrials.gov were searched through January 1, 2025. Clinical trials reporting outcomes such as overall response rate (ORR), progression-free survival (PFS), overall survival (OS), and adverse events (AEs) in RT patients treated with BTK inhibitors were included.

**Results:**

Seven studies (six clinical trials and one case series) comprising 220 patients were included. Monotherapy with pirtobrutinib and acalabrutinib showed ORRs of 50% and 40%, respectively. Combination regimens such as zanubrutinib plus tislelizumab and ibrutinib plus nivolumab demonstrated ORRs ranging from 41.6% to 65%, with improved outcomes in treatment-naïve patients. Safety profiles were generally manageable, though grade ≥3 AEs, particularly cytopenias and infections, were common. Risk of bias was moderate to serious across studies due to non-randomized designs and small sample sizes.

**Conclusion:**

BTK inhibitor–based therapies show promising efficacy in patients with RT, particularly in combination with immunotherapeutic agents. While monotherapy may offer a tolerable option for frail patients, combination regimens may improve outcomes in select populations. Larger, randomized controlled trials are needed to better define the role of BTK inhibition in this high-risk disease.

## Introduction

Richter’s transformation (RT) is an aggressive lymphoma in patients with previous or concomitant diagnosis of chronic lymphocytic leukemia (CLL) or small lymphocytic lymphoma (SLL) (1). It occurs in 2-10% of patients diagnosed with CLL and is considered as a phenotypic transdifferentiating phenomenon in which patients develop a diffuse large B-cell lymphoma (DLBCL, in 95–99% of the cases) or less commonly Hodgkin’s lymphoma (0.5-5% of the cases) (1–3). The annual incidence rate of RT in patients with CLL has been estimated around 0.5–1%.

Previous works investigating RT identified that transformation to DLBCL is commonly a linear evolutionary phenomenon arising from a dominant CLL clone, and it is a genetically distinct entity from *de novo* DLBCL with a unique biology (4). Somatic alterations in tumor suppressor genes, cell cycle and genes involved in proliferation pathways such as *TP53, NOTCH1, MYC, CDKN2A* are the main genetic clues of DLBCL-RT, which can explain the aggressive nature of the disease (5).

The characteristic presentation of RT are new onset B symptoms (fever, night sweats, weight loss), pronounced increase in lymphadenopathy, significant elevations of lactate dehydrogenase (LDH) and associated multiorgan dysfunction from invasive or obstructive process (3). Extranodal involvement can be present especially in gastrointestinal (GI) tract, bone marrow, central nervous system or skin.

RT should be suspected in patients with CLL who present with sudden clinical deterioration, constitutional symptoms including B symptoms, an asymmetric and rapid growth of lymph nodes. Signs of extranodal involvement may manifest as early satiety, GI bleeding, rash, pathologic fractures, headache, blurred vision or dyspnea (6). On laboratory tests the common findings are cytopenias, elevated LDH, and less frequently hypercalcemia. Clinical risk factors for the RT include advanced stage of CLL, bulky lymphadenopathy or hepatosplenomegaly, low platelet count, elevated beta-2 microglobulin, prior CLL therapy involving purine analogs and alkylating agents, a high number of lines of therapy for CLL (2).

The use of 18F-FDG PET/CT plays a critical role in the evaluation of suspected Richter’s transformation. While PET/CT is not routinely recommended in the standard monitoring of chronic lymphocytic leukemia (CLL) due to its generally indolent nature, it becomes a highly valuable tool when RT is suspected. Increased FDG uptake may help differentiate RT from progressive CLL, and studies have shown that a standardized uptake value (SUV) >10 is strongly associated with RT. PET/CT also assists in identifying the most metabolically active lesion for biopsy, thereby increasing diagnostic yield and avoiding sampling error. In patients presenting with constitutional symptoms, rapidly enlarging lymphadenopathy, or signs of extranodal involvement, PET/CT should be considered as part of the diagnostic workup (7).

The gold standard for the diagnosis of RT is biopsy with histologic documentation of the disease. Fine needle biopsy is not recommended considering that it may not illustrate the whole lymph node structure (5). RT is often limited to one single lesion during evolution, this makes it important to choose where to perform biopsy. FDG PET/CT assists with the biopsy of the most fluorodeoxyglucose-avid lymph node (7).

RT which is clonally unrelated to CLL demonstrates improved outcomes similar to *de novo* DLBCL with median survivals of 62.5 months, while clonally related RT has OS of 14 months for clonally related RT (3). The median overall survival of patients diagnosed with RT rounds 12 months; however, this expectancy decreases with the presence of other risk factors or previous exposure to treatment regimens (8). Multiagent chemoimmunotherapy regimens, such as rituximab plus cyclophosphamide, doxorubicin, vincristine, and prednisone (R-CHOP), are the most commonly used initial therapy. Although intensified regimens such as DA-R-EPOCH have been explored, retrospective analyses suggest they have not consistently demonstrated superior outcomes and may carry increased toxicity in this patient population (9). The outcomes with chemoimmunotherapy are far poorer than observed with the same regimens used in *de novo* diffuse large B-cell lymphoma. Notably, patients with treatment-naïve CLL at the time of RT have superior survival compared to those previously treated for CLL (5, 10).

Bruton’s tyrosine kinase (BTK) inhibitors, such as ibrutinib, acalabrutinib, and zanubrutinib, have revolutionized the treatment of CLL by targeting B-cell receptor signaling, a key driver of CLL pathogenesis. However, their role in RT remains less defined. While BTK inhibitors demonstrate efficacy in delaying CLL progression and reducing RT risk in treatment-naïve patients, their utility in established RT, particularly in relapsed/refractory cases, is an area of active investigation. Emerging evidence suggests that BTK inhibition may be an effective therapy for RT, but response durability and safety profiles in this aggressive setting are poorly characterized. This article reviews contemporary data on BTK inhibitor-based therapies in RT, evaluating their safety and efficacy across treatment-naïve and relapsed/refractory populations, and explores strategies to optimize their use within multimodal regimens.

## Methods

A systematic literature review was conducted in accordance with PRISMA (11) and Cochrane (12) guidelines.

### Search strategy

A comprehensive search was performed on PubMed, EMBASE, and ClinicalTrials.gov with the keywords, “Richter’s Transformation” and “Bruton’s Tyrosine Kinase Inhibitors.” The literature search was performed from the beginning of the data until 1 January 2025. The PICO framework was used to perform this literature search.

### Inclusion and exclusion of articles

All the clinical trials providing safety (adverse effects) and efficacy (overall response rate, progression-free survival, overall survival) data on BTK inhibitors in RT were included. All the review articles, case reports, preclinical studies, and clinical studies irrelevant to BTKi drugs or RT were excluded. All the clinical trials without any safety or efficacy outcomes were also excluded.

### Study selection

**Figure 1:**
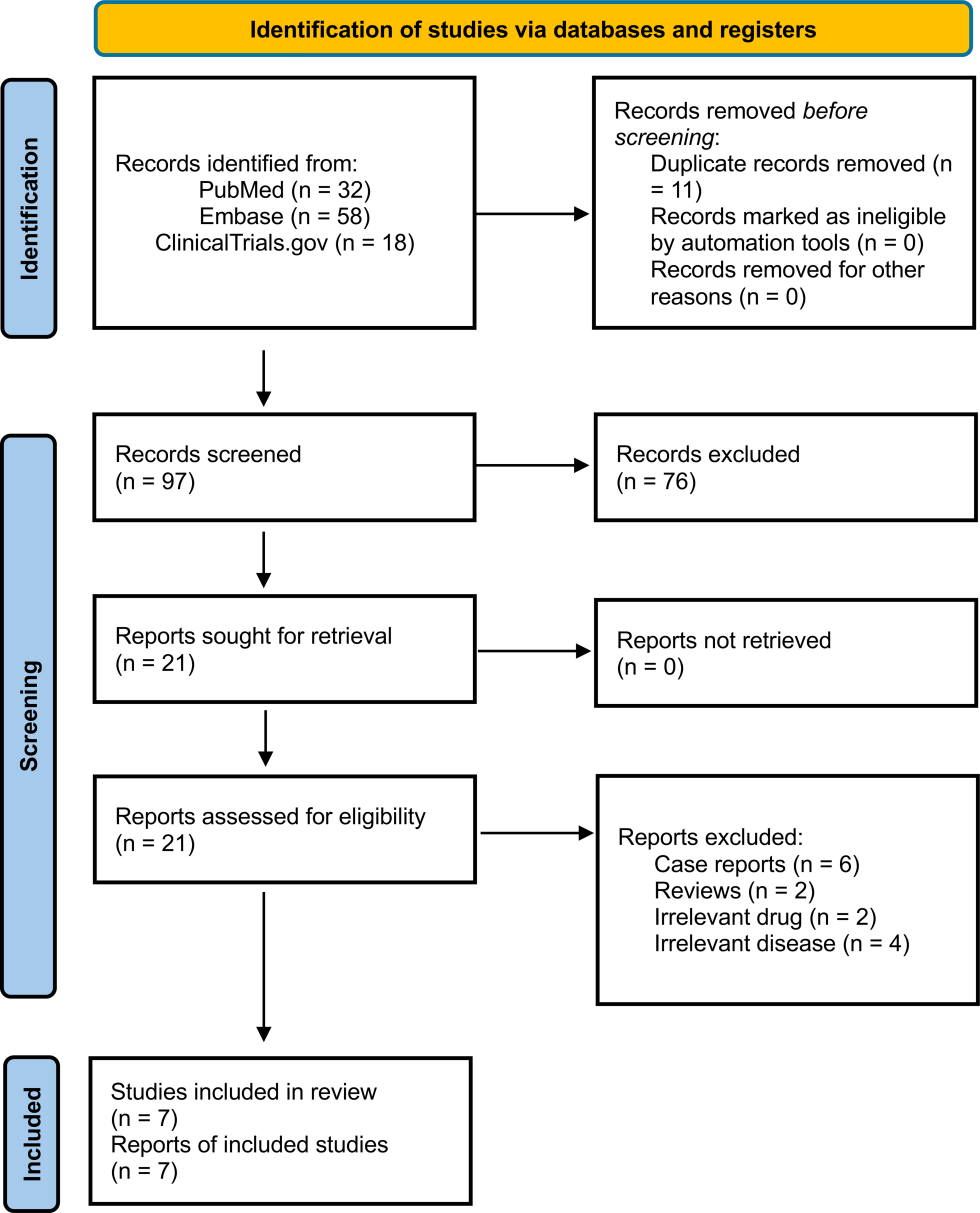
PRISMA 2020 flow diagram of study identification, screening, and inclusion process.

Article screening was conducted independently by two authors (CDD and AM) using pre-specified inclusion criteria. Discrepancies were resolved through discussion with a third reviewer (AS). The study selection process is illustrated in the PRISMA flow diagram (**Figure 1**).

### Data extraction

Two authors (CDD and AM) extracted the relevant data for the baseline characteristics of the included studies (treatment drug and dose, median age, number of treatment-naïve patients, patient that received any prior therapy for RT and patient that received only prior treatment with BTKi for RT), efficacy outcomes (overall response rate, progression-free survival, overall survival), and adverse events (any adverse events, ≥grade 3 adverse effects).

### Risk of bias assessment

ROB was conducted by using the ROBINS-I tool by two researchers (FA and BCAF).

## Results

From databases, 32 articles were identified from PubMed, 58 articles were identified from Embase, and 18 from the registry of clinicaltrials.gov. After careful screening of the articles, six non-randomized clinical trials and one real world study (*N* = 220) (13–19) were included.

### Risk of bias

Risk of bias was calculated using the ROBINS-I tool. Included studies were non-randomized, single-arm clinical trials and retrospective case series and the overall risk was judged to be serious (**Table 1**).

**Table 1:** Summary of risk of bias across included studies (ROBINS-I assessment).

Study	Bias due to confounding	Bias in selection of participants into the study	Bias in classification of interventions	Bias due to deviations from intended interventions	Bias due to missing data	Bias in measurement of outcomes	Bias in selection of the reported result
Wierda et al. [2024] (17)	Serious	Moderate	Low	Moderate	Moderate	Moderate	Low to moderate
Eyre et al. [2021] (14)	Serious	Moderate	Low	Low	Low	Moderate	Moderate
Al-Sawaf et al. [2024] (18)	Serious	Moderate	Low	Low to moderate	Low	Moderate	Low to Moderate
Jain et al. [2023] (16)	Serious	Moderate	Low	Low	Low	Moderate	Low
Younes et al. [2019] (13)	Serious	Moderate	Low	Moderate	Low	Moderate	Moderate
Stephens et al. [2022] (15)	Serious	Moderate	Low	Moderate	Low	Moderate	Moderate
Pongas et al. [2025] (19)	Serious	Serious	Low	Moderate	Low	Moderate	Moderate

All studies were designed as single-arm and lacked a control group. The included patients were heterogeneous with variable prior therapies, clonality and disease burden. Additionally, none of these studies reported blinded or independent outcome assessment. While interventions were well defined and data completeness was adequate, studies demonstrated serious risk for confounding and moderate risk in participant selection and outcome measurement.

### Efficacy and safety of BTKi

In total, 220 adult patients were included across six clinical trials and one case series evaluating BTK inhibitor-based therapies for Richter’s transformation. Of these, 82 patients (37.2%) received pirtobrutinib monotherapy, while 25 patients (11.3%) were treated with acalabrutinib monotherapy. Combination regimens included zanubrutinib plus tislelizumab in 48 patients (21.8%), platinum-based chemotherapy with BTK inhibitors in 12 patients (5.4%), ibrutinib plus nivolumab in 44 patients (20%), and ibrutinib plus selinexor in 9 patients (4%). Regarding treatment history, 78 patients (35.4%) were treatment-naïve, while 142 patients (64.5%) had relapsed or refractory disease. Notably, 98 patients (44.5%) had previously been treated with BTK inhibitors. Baseline characteristics for all patients are summarized in **Table 2**.

**Table 2:** Baseline characteristics of patients in included studies.

Trial	NCT	Drug therapy	Trial phase	Number of participants	Median age (Range)	Treatment naïve patients n (%)	Prior treatments n (%)	Prior BTKi n (%)
Wierda et al. [2024] (17)	NCT03740529	Pirtobrutinib	Phase I/II	82	67 (59–72)	8 (9.7%)	74 (90.2%)	61(74.4%)
Eyre et al. [2021] (14)	NCT02029443	Acalabrutinib	Phase I/II	25	66 (58–73)	11 (44%)	14 (58.3%)	NR
Al-Sawaf et al. [2024]	NCT04271956	Tislelizumab plus zanubrutinib	Phase II	48	67 (45–82)	38 (79.1%)	10 (20.8%)	24 (50%)
Jain et al. [2023]	NCT02420912	Nivolumab plus ibrutinib	Phase II	24	64.5 (47–88)	14 (58.3%)	10 (41.6%)	13 (54.1%)
Younes et al. [2019] (13)	NCT02329847	Nivolumab plus ibrutinib	Phase I/II	20	67·5 (56–70)	0	20 (100%)	0
Stephens et al. [2022] (15)	NCT02303392	Selinexor plus ibrutinib	Phase I	9	62 (59–78)	0	9 (100%)	0
Pongas et al. [2025]		RICE plus BTKi	Case series	12	61 (53–71)	7 (58.3%)	5 (41.6%)	NR

NCT, National Clinical Trial; BTKi, Bruton Tyrosine Kinase inhibitor, RICE:

1. Efficacy of BTKi as monotherapy (Pirtobrutinib and Acalabrutinib)

In a phase I/II clinical trial conducted by Wierda et al. (N = 82), Pirtobrutinib—a non-covalent BTK inhibitor—was evaluated in patients with Richter’s transformation (RT), including 74 with relapsed/refractory disease and 8 treatment-naïve cases. The overall response rate (ORR) was 50%, comprising 30 patients with a partial response and 11 with a complete response. The median duration of response was 7.4 months. Median progression-free survival (PFS) and overall survival (OS) were 3.7 and 12.5 months, respectively. Subgroup analysis revealed an ORR of 65.2% in treatment-naïve patients, 48.6% in previously treated patients, and 62.5% in those with prior BTK inhibitor exposure. Median PFS was 10.1 months for treatment-naïve patients and 3.6 months for those with relapsed/refractory disease.

In a phase I/II clinical trial by Eyre et al. (N = 25), acalabrutinib monotherapy was evaluated in patients with RT. The cohort included 11 treatment-naïve patients and 14 with prior therapy. The ORR was 40%, with 8 patients achieving a partial response and 2 achieving a complete response. The median duration of response was 6.2 months, and the median PFS was 3.2 months. Subgroup analysis showed an ORR of 36% in treatment-naïve patients, 43% in relapsed/refractory cases, and 25% in those previously treated with a BTK inhibitor. Median PFS was 3.2 months for treatment-naïve patients and 2.0 months for relapsed/refractory patients.

2. Efficacy of BTKi as part of a combination regimen (Pirtobrutinib and Acalabrutinib)

18 investigated the efficacy of a combination regimen of tislelizumab and zanubrutinib in a Phase II trial involving 48 patients with Richter’s transformation. The majority (n = 38) were treatment-naïve, while 10 had received prior therapy; notably, 24 patients had been previously treated with a BTK inhibitor. The study reported an ORR of 58.3%, including 19 partial responses and 9 complete responses. Median PFS was 10 months, and the OS rate at 12 months was 74.7%. In subgroup analyses, ORR was 57.9% in treatment-naïve patients, 60% in those with relapsed/refractory disease, and 47.3% among patients with prior BTK inhibitor exposure.

Several clinical trials have explored combination therapies involving ibrutinib in the treatment of RT. In a Phase II trial conducted by 16. (N = 24), the combination of ibrutinib and the PD-1 inhibitor nivolumab was evaluated. Of the enrolled patients, 14 were treatment-naïve, 10 had received prior lines of therapy, and 13 had prior exposure to BTK inhibitors. The ORR was 41.6%, with 2 patients achieving a partial response and 8 achieving a complete response. The median duration of response was 15 months, and median OS was 13 months. Subgroup analysis revealed an ORR of 50% in treatment-naïve patients, 30% in those with relapsed/refractory disease, and 23% in those previously treated with BTK inhibitors. The median OS was notably longer in treatment-naïve patients (24.1 months) compared to relapsed/refractory patients (9.1 months).

Similarly, Younes et al. assessed the efficacy of the nivolumab–ibrutinib combination in a Phase I/IIa trial involving 20 patients with relapsed/refractory RT. The study reported an ORR of 65%, including 11 partial responses and 2 complete responses. The median duration of response was 6.9 months, with a median progression-free survival (PFS) of 5 months and a median OS of 10.3 months.

In a smaller Phase I trial (N = 9), Stephens et al. evaluated ibrutinib in combination with selinexor. The ORR was modest at 11.1%, while 55.5% of patients achieved stable disease.

19 conducted a retrospective case series involving 12 patients treated with R-ICE chemotherapy in combination with a BTK inhibitor. This regimen yielded an ORR of 83%, with 58% of patients achieving a complete response. The median PFS and OS were 54 months and 57 months, respectively. Among BTK inhibitor–naïve patients, the ORR was 86%, compared to 80% in those with prior BTK inhibitor exposure. Interestingly, the 2-year OS was 40% in the BTKi-naïve group and 60% in the BTKi-pretreated group.

3. Safety of BTK inhibitors

In the trial of pirtobrutinib, 94% (77 of 82) of patients experienced treatment-emergent AEs, with 60% (49 of 82) experiencing grade 3 or higher events. The most frequently reported AEs (any grade) included dyspnea, pyrexia, diarrhea, and contusion (15 patients each). Grade ≥3 AEs occurring in more than 15% of patients included neutropenia (23%), infections (26%), and thrombocytopenia (11%). Despite the high incidence of adverse events, treatment discontinuation due to toxicity was low, reported in only 6% of patients.

In the acalabrutinib trial, commonly reported AEs of any grade included headache (41%), diarrhea (35%), anemia (31%), fatigue (24%), arthralgia (17%), and back pain (17%). Grade ≥3 AEs occurred in 62% of patients, with anemia and neutropenia (14% each), hypercalcemia (10%), and back pain (10%) being the most prevalent. Notably, there were no treatment discontinuations due to AEs.

In the tislelizumab and zanubrutinib combination study, nearly all patients (98.2%) experienced at least one AE of grade ≥1. The most frequent events were gastrointestinal disorders, occurring in 56.1% of patients. Four patients discontinued treatment due to adverse events.

For the nivolumab–ibrutinib combination, Jain et al. reported that 16.6% of patients experienced grade ≥3 AEs, with one treatment discontinuation attributed to toxicity. In contrast, the trial by Younes et al. found diarrhea (33%), neutropenia (31%), and fatigue (26%) as the most common AEs of any grade. Grade 3–4 AEs included neutropenia (28%) and anemia (23%).

Overall, while AEs were frequently observed across these studies, the safety profiles were generally manageable, with relatively low discontinuation rates. Summary of safety and efficacy of BTK inhibitors are summarized in **Table 3**. **Table 4** provides an overview of ongoing clinical trials assessing BTK inhibitor–based therapies in patients with Richter’s transformation.

**Table 3:** Summary of efficacy and safety in studies of BTK inhibitor–based therapies for Richter’s transformation.

Trial	Drug therapy	ORR				Median PFS			Median OS			Grade 3 and more AE	Discontinuation due to Aes
		All	Treatment naive	Relapsed/refractory	Prior BTKi	All	Treatment naive	Relapsed/refractory	All	Treatment naive	Relapsed/refractory		
Wierda et al [2024] (17)	Pirtobrutinib	41/82 (50%)	5/8 (62.5%)	34/74 (48-6%)	28/61 (62-5%)	3.7 (2.7- 4-9)	10.1 (1.3 16.8)	3.6 (2.4- 4-6)	12.5 (6.9- 20-5)	13.25 (2.6 -18.3)	11.8 (6.9- 19-5)	59.7%	6%
Eyre et al [2021] (14)	Acalabrutinib	10/25 (40%)	4/11 (36%)	6/14 (43%)	3/12 (25%)	3.2 (1.8- 4-0)	3-2 (1–6– 3–7)	2.0 (1–7– 11–1)	NR	NR	NR	84%	0
Al-Sawaf et al [2024]	Tislelizumab plus zanubrutinib	28/48 (58.3%)	22/38 (57.9%)	11/19 (60%)	9/19 (47.3%)	10 (3.8- 16.3)	10	Not reached	NR	NR	NR	NR	6%
Jain et al [2023]	Nivolumab plus ibrutinib	10/24 (41.6%)	7/14 (50%)	3/10 (30%)	3/13 (23%)	NR	NR	NR	13	9.1	24.1	16.6%	0
Younes et al [2019]	Nivolumab plus ibrutinib	13/20 (65%)	NR	NR	NR	NR	NR	NR	10.3	NR	NR	35%	45%
Stephens et al [2022] (15)	Selinexor plus ibrutinib	1/9 (11.1%)	NR	NR	1/9 (11.1%)	NR	NR	NR	NR	NR	NR	24%	14%
Pongas et al [2025]	RICE plus BTKi	10/12	NR	NR	80%	54	NR	NR	57	NR	NR	NR	NR

**Table 4:** Ongoing clinicals assessing the safety and efficacy of BTK inhibitor-based therapies for Richter’s transformation.

NCT	Drug	Phase	N	Outcomes	End date
NCT04271956	Zanubrutinib + Tislelizumab with or without Sonrotoclax	Phase II	83	Safety and Efficacy	2026
NCT06735664	Zanubrutinib + Odronextamab	Phase I	23	Safety and Efficacy	2027
NCT06043674	Glofitamab + pirtobrutinib	Phase II	10	Safety and Efficacy	2033
NCT03162536	Nemtabrutinib	Phase I/II		Safety and Efficacy	2025
NCT05672173	Lisocabtagene Maraleucel, Nivolumab and Ibrutinib	Phase II	20	Safety and Efficacy	2025
NCT06863402	Nemtabrutinib + Pembrolizumab	Phase II	32	Safety and Efficacy	2030
NCT05388006	Acalabrutinib, Venetoclax and Durvalumab	Phase II	30	Safety and Efficacy	2027

## Discussion

RT is associated with a historically poor prognosis and limited therapeutic options, and it remains a clinical challenge in the management of patients with CLL. BTK inhibitors have emerged as a potential therapeutic avenue for RT considering their established efficacy in CLL. However, their role in the treatment of RT is still evolving. In this review we aimed to assess the current evidence surrounding BTK inhibitor–based strategies in both monotherapy and combination settings for the management of RT.

BTKis have significantly transformed the treatment paradigm in CLL. First generation agents such as ibrutinib have demonstrated durable efficacy in both treatment-naïve and relapsed/refractory CLL, with overall response rates exceeding 80%. Long-term follow-up showed superior progression-free and overall survival compared to traditional chemoimmunotherapy (20). In contrast, the activity of BTK inhibitors in diffuse large B-cell lymphoma (DLBCL) is more limited and appears to be subtype-specific. Clinical studies have shown modest responses primarily in the activated B-cell (ABC) subtype, while the germinal center B-cell (GCB) subtype exhibits minimal benefit (21).

Compared to traditional chemoimmunotherapy regimens such as R-CHOP (rituximab, cyclophosphamide, doxorubicin, vincristine, and prednisone) or R-EPOCH (rituximab, etoposide, prednisone, vincristine, cyclophosphamide, and doxorubicin), which yield modest response rates and a median overall survival of less than 12 months in Richter’s transformation, BTK inhibitor–based therapies have shown comparable or superior efficacy with improved tolerability, particularly in frail or heavily pretreated patients.

Our analysis of seven non-randomized studies including 220 patients demonstrates that BTK inhibitors can induce meaningful responses in RT. However, the outcomes vary significantly depending on treatment context and patient characteristics. Pirtobrutinib, a non-covalent BTK inhibitor, demonstrated an ORR of 50% in a heavily pretreated cohort, with a median OS of 12.5 months, which is comparable or superior to historical data for RT treated with chemoimmunotherapy. Response rates were high in both treatment-naïve patients (65.2%) and those with prior BTKi exposure (62.5%), which highlights the therapeutic potential of pirtobrutinib even in pretreated populations.

Similarly, acalabrutinib monotherapy showed moderate efficacy, with a 40% ORR and a median PFS of 3.2 months. The outcomes were more modest with acalabrutinib; however, this agent was generally well tolerated, with no treatment discontinuations due to adverse events. These data suggest a role for BTK inhibitors as a tolerable option for select patients with RT, particularly those who are frail or ineligible for cytotoxic chemotherapy.

Combination strategies were also assessed and have shown promise in enhancing efficacy. The combination of tislelizumab and Zanubrutinib demonstrated an ORR of 58.3% and a 12-month OS rate of 74.7%. This finding suggests a potential synergy between BTK inhibition and immune checkpoint blockade. The combination of ibrutinib and nivolumab produced ORRs ranging from 41.6% to 65% across two studies, with longer median OS in treatment-naïve patients, which is 24.1 months compared to 9.1 months in patients with relapsed/refractory disease. These findings support a tailored approach to therapy based on treatment history and patient fitness and underscore the potential of integrating BTK inhibition with immunomodulatory agents.

However, not all combinations showed high efficacy. The ibrutinib–selinexor regimen had limited activity (ORR 11.1%), highlighting the variability in benefit among different combinations. On the other hand, a small retrospective case series evaluating RICE plus BTK inhibitors showed impressive response rates (ORR 83%, median OS 57 months), particularly in BTKi-naïve patients, suggesting that combination with cytotoxic chemotherapy may remain a viable option in selected individuals.

In terms of safety, regimens containing BTK inhibitors had generally manageable toxicity profiles. While AEs were frequent—particularly cytopenias, infections, and gastrointestinal symptoms—most studies reported low discontinuation rates. Grade ≥3 AEs were most common with pirtobrutinib and combination regimens, but treatment-limiting toxicity was uncommon. These findings underscore the importance of AE monitoring, especially in combination protocols, but overall support the feasibility of BTKi-based regimens even in a heavily pretreated RT population.

There are several limitations of the current evidence. The included studies were all early-phase or non-randomized trials, with relatively small patient numbers and heterogeneous populations. Therefore, cross-trial comparisons should be made with caution. Molecular correlates such as TP53 mutation status, clonal relationship to CLL, and other biomarkers were not uniformly reported. This limits the ability to draw conclusions about predictive factors for response and analyze which patient population may benefit from a particular medication regimen. Lastly, follow-up durations were relatively short, and long-term durability of response remains uncertain in most cohorts.

Despite these limitations, the current data provide a rationale for the continued investigation of BTK inhibitors in RT. Future directions include the design of randomized controlled trials to define the comparative efficacy of BTKi-based regimens, the integration of novel agents such as BCL2 inhibitors or bispecific antibodies, and the exploration of biomarkers to guide patient selection. Assessing the outcomes stratified by clonal relationship to CLL, molecular profile, and prior treatment history may provide more insights into therapeutic tailoring.

In conclusion, BTK inhibitors, both as monotherapy and in combination regimens, represent an emerging and promising option for patients with Richter’s transformation. While outcomes remain heterogeneous, especially in the relapsed/refractory setting, these therapies offer a favorable safety profile and a non-chemotherapy alternative that may be particularly valuable in frail or heavily pretreated patients. As data from ongoing trials mature, the role of BTK inhibitors in the evolving RT treatment landscape will become clearer and may ultimately contribute to improved outcomes in this historically intractable disease.
